# Functional Divergence and Convergent Evolution in the Plastid-Targeted Glyceraldehyde-3-Phosphate Dehydrogenases of Diverse Eukaryotic Algae

**DOI:** 10.1371/journal.pone.0070396

**Published:** 2013-07-30

**Authors:** Daniel Gaston, Andrew J. Roger

**Affiliations:** 1 Department of Biochemistry and Molecular Biology, Dalhousie University, Halifax, Nova Scotia, Canada; 2 Centre for Comparative Genomics and Evolutionary Bioinformatics, Dalhousie University, Halifax, Nova Scotia, Canada; California State University Fullerton, United States of America

## Abstract

**Background:**

Glyceraldehyde-3-phosphate dehydrogenase (GAPDH) is a key enzyme of the glycolytic pathway, reversibly catalyzing the sixth step of glycolysis and concurrently reducing the coenzyme NAD^+^ to NADH. In photosynthetic organisms a GAPDH paralog (Gap2 in Cyanobacteria, GapA in most photosynthetic eukaryotes) functions in the Calvin cycle, performing the reverse of the glycolytic reaction and using the coenzyme NADPH preferentially. In a number of photosynthetic eukaryotes that acquired their plastid by the secondary endosymbiosis of a eukaryotic red alga (Alveolates, haptophytes, cryptomonads and stramenopiles) GapA has been apparently replaced with a paralog of the host’s own cytosolic GAPDH (GapC1). Plastid GapC1 and GapA therefore represent two independent cases of functional divergence and adaptations to the Calvin cycle entailing a shift in subcellular targeting and a shift in binding preference from NAD^+^ to NADPH.

**Methods:**

We used the programs FunDi, GroupSim, and Difference Evolutionary-Trace to detect sites involved in the functional divergence of these two groups of GAPDH sequences and to identify potential cases of convergent evolution in the Calvin-cycle adapted GapA and GapC1 families. Sites identified as being functionally divergent by all or some of these programs were then investigated with respect to their possible roles in the structure and function of both glycolytic and plastid-targeted GAPDH isoforms.

**Conclusions:**

In this work we found substantial evidence for convergent evolution in GapA/B and GapC1. In many cases sites in GAPDHs of these groups converged on identical amino acid residues in specific positions of the protein known to play a role in the function and regulation of plastid-functioning enzymes relative to their cytosolic counterparts. In addition, we demonstrate that bioinformatic software like FunDi are important tools for the generation of meaningful biological hypotheses that can then be tested with direct experimental techniques.

## Introduction

**Figure 1:**
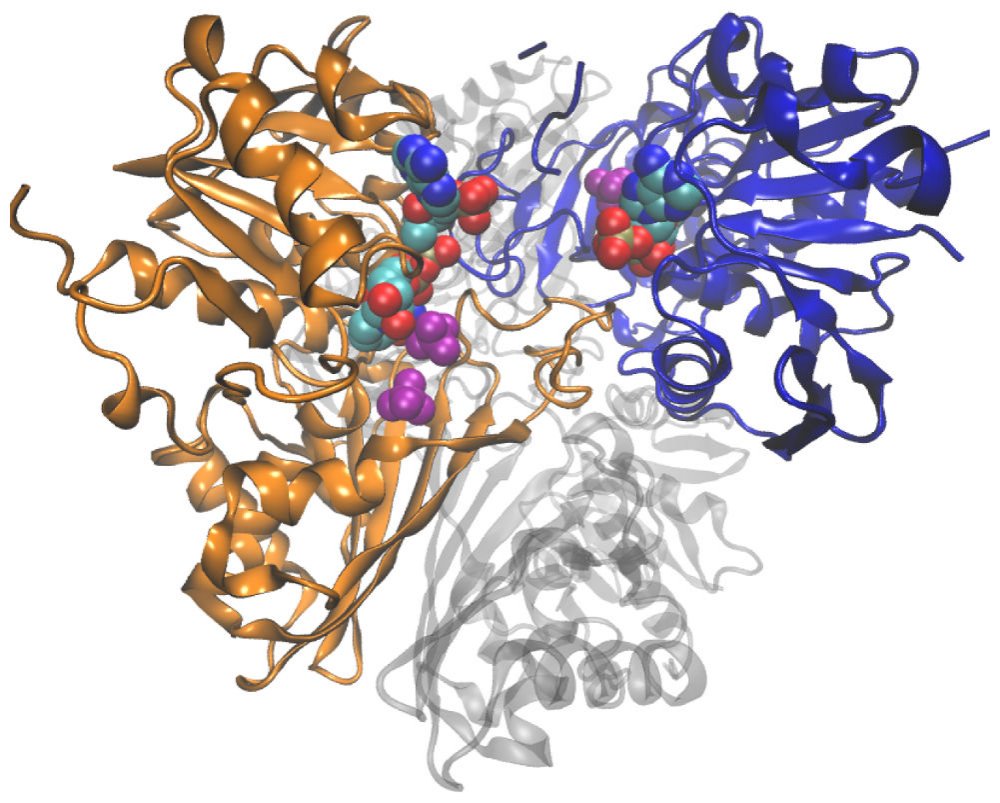
Quaternary structure of the GAPDH A_2_B_2_ heterotetramer (PDB: 2PKQ) from *Spinacia oleracea* (spinach). Crystallographic subunits O (GapB) and R (GapA) are show in blue and orange respectively. Bound NADPH in the co-enzyme binding domain drawn as a van der Waals space-filling model coloured by element type according to the Visual Molecular Dynamics (VMD) program. Sulfate ions in the Ps and Pi in purple, also as space-filling models. The O and R subunit are related by the R axis of symmetry. The P and Q subunits are shown in the background.

Cytosolic glyceraldehyde-3-phosphate dehydrogenase (GAPDH) reversibly catalyzes the sixth step of glycolysis, the conversion of glyceraldehyde 3-phosphate to D-glycerate 1,3-bisphosphate, reducing the coenzyme NAD^+^ to NADH in the process [1,2]. Functional GAPDH is a homotetramer, with each monomer composed of two domains: the N-terminal coenzyme binding domain and the C-terminal catalytic domain. The catalytic domain contains the P_s_ and P_i_ sites, which bind the C_(3)_ phosphate of the substrate and the inorganic phosphate ion respectively during the phosphorylation step carried out by the enzyme [3]. Another important structural feature, the S-loop, folds over in close proximity to the bound cofactor (Figure 1).

The Archaeplastida (land plants, green algae, red algae, and glaucophytes) are eukaryotes that have acquired their chloroplasts via the primary endosymbiosis of a cyanobacterium [4–6] and have a plastid-targeted GAPDH, homologous to the Gap2 of cyanobacteria [7]. This GAPDH is active in the Calvin cycle where it preferentially carries out the reverse of the glycolytic reaction [8]. Unlike its glycolytic homologs, the plastid-targeted GAPDH (GapA) has dual coenzyme specificity with a marked preference for binding (and oxidizing) NADPH in the Calvin cycle reaction. GapB is a land plant specific duplicate of GapA [9] with a C-terminal extension consisting of approximately 30 amino acids that is homologous to the CP12 regulatory enzyme [10]. Plastid targeted forms of GAPDH can also participate in the oxidative pentose phosphate pathway (OPP) [11,12]; because of these dual-roles, NADPH-dependent GAPDH is differentially regulated during light and dark cycles. This regulation is thioredoxin-mediated through CP12, a small intrinsically unstructured protein that forms a protein complex with both GAPDH and ribulophosphokinase (RPK) [13–15].

Crystal structures for the A_2_B_2_ tetramer of streptophytes, and mutant A_4_ tetramers [16,17], have been solved, including fragments of the C-terminal extension of GapB that is homologous to CP12, yielding insights into the structural determinants of cofactor discrimination and its CP12-mediated regulation [18,19]. Under dark conditions CP12 is oxidized, resulting in the formation of two internal disulphide bridges which reduce the overall disorder of CP12 and increase the helical content, allowing CP12 to act as a scaffold in the formation of the GAPDH/CP12/PRK supramolecular complex. Under light-conditions thioredoxin reduces CP12, which releases PRK from the complex [11,12,20,21], GAPDH is released and fully activated in the presence of the substrate 1,3-biphosphoglycerate and when NADPH to NADP^+^ ratios shift in favour of NADPH [13,22].

**Figure 2:**
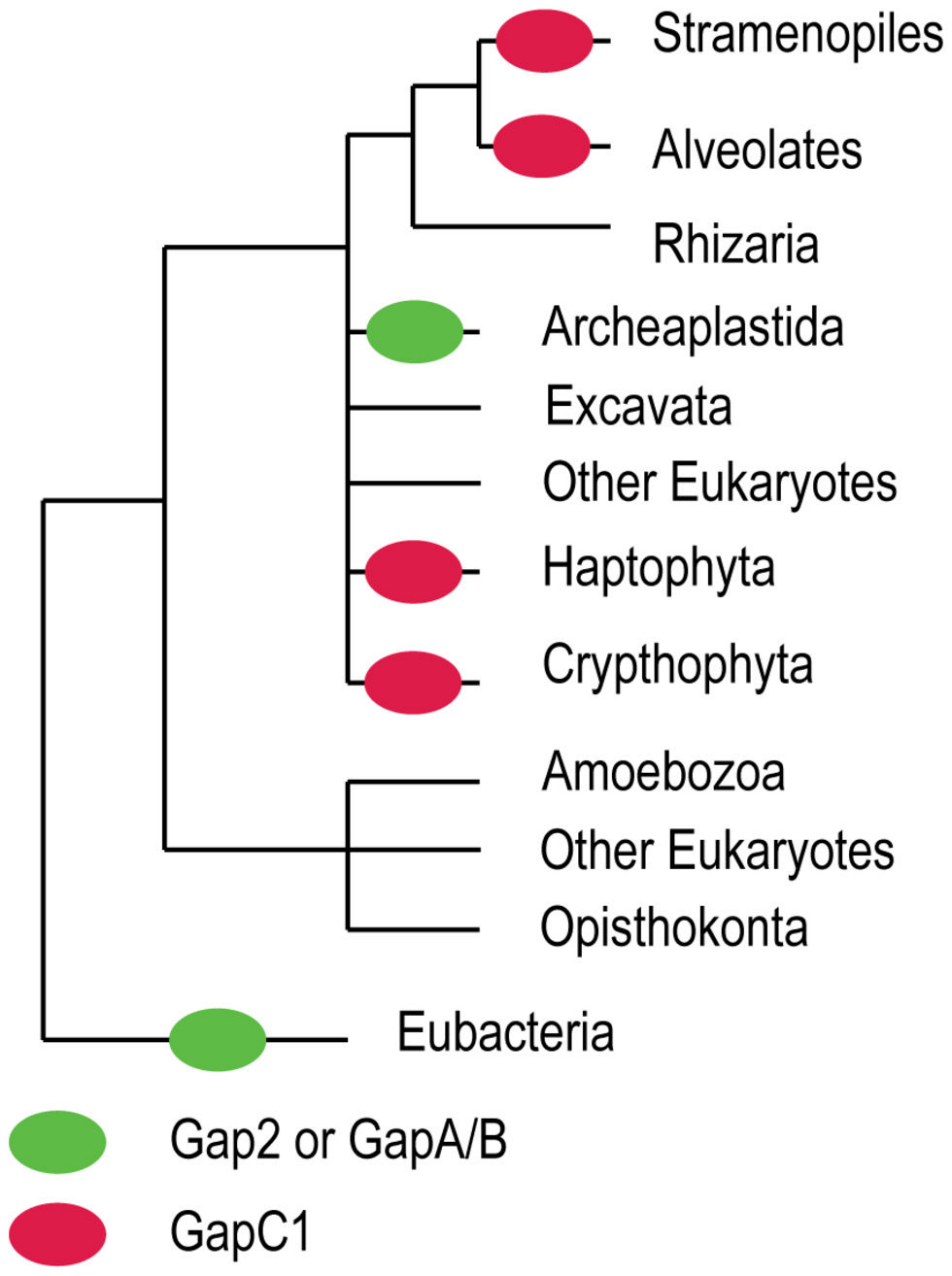
A simplified representation of the organismal phylogeny for relevant taxa in this study. Green oval represents the endosymbiotic event in the ancestor of the Archaeplastida (Green Algae, Red Algae, Glaucophytes, Land Plants, etc) that gave rise to the modern chloroplast. Gap2 of cyanobacteria became the GapA of the Archaeplastida, with a further gene duplication event in land plants giving rise to GapB. Many of the relationships between eukaryotic supergroups are currently unresolved, and are represented with multifurcations. Indicated with red ovals are lineages that contain the GapC1 gene. While the gene itself is monophyletic, it is unlikely that the lineages that harbour the red-algal derived plastids they are found in represent a clade, with the Haptophytes and Cryptophytes having uncertain affiliation but probably not with the Stramenopiles and Alveolata as once was thought.

Outside of the Archaeplastida, photosynthetic eukaryote lineages exist that originally acquired their chloroplasts via secondary endosymbiosis of primary eukaryotic algae. In many of these lineages, a duplicate copy of cytosolic GAPDH (CapC1) was re-targeted to the chloroplast that functionally replaces GapA [23–25]. These enzymes also feature dual coenzyme specificity with a preference for NADPH when functioning in the Calvin cycle. The Chromalveolata is a proposed monophyletic ‘super-group’ of microbial eukaryotes comprised of the stramenopiles, haptophytes, cryptophytes, and alveolates [26–28]. Several lineages within each of these monophyletic groups contain a red-algal derived plastid of secondary endosymbiotic origin (For review, see 29) The presumed rarity of successful secondary endosymbiotic integration, along with several molecular phylogenies, including that of GapC1, have been used as support for this ‘chromalveolate hypothesis’ [23,30–33]. However, recent work by Takishita and colleagues [34] has shown that extensive LGT within the GapC1 group produces a phylogeny inconsistent with the presumed organismal phylogeny and may make its usefulness for determining chromalveolate monophyly less clear. In addition, various phylogenetic studies recover support for alternative groupings that modify or reject chromalveolate monophyly [35–39] or favour alternative models, such as multiple serial eukaryote-to-eukaryote enodymbioses [40]. The current view of eukaryotic phylogeny is shown in Figure 2, with GapA/B and GapC1 lineages indicated. Regardless of whether the chromalveolate taxa truly form a monophyletic group, the transition of GapC1 from a cytosolic, NAD^+^-binding GAPDH to a plastid-targeted, NADPH-dependent GAPDH appears to have occurred only once, and GapC1 sequences are monophyletic. To avoid confusion, we will henceforth refer to the various monophyletic algal lineages that contain that GapC1 sequences by name rather than using the contentious term ‘chromalveolate’. While the evolutionary history of the red-algal derived plastid and their host organisms is likely very complex, we expect there to have been relatively uniform evolutionary pressures on GAPDH function within these plastids. On the other hand, because the host organisms inhabit widely varying environments and have different metabolic properties, there appear to be many differences in terms of the regulation of GapC1 function (discussed below).

A large scale comparison of GAPDH and PRK regulation in algae, including the GapC1 containing groups [41], has shown variation within the latter group and between GapC1-containg algae and archaeplastid taxa with canonical GapA plastid-targeted sequences. Unfortunately, many of the species examined in the regulation studies are not the same species for which we have molecular sequence data for GapC1. In the diatoms *Odontella* *sinensis* [42], *Thalassiosira pseudonana*, and *Phaeodactylum* *tricornutum* [43] the entire oxidative pentose phosphate pathway (OPPP) appears to be cytosol localized instead of occurring in the plastid as in most other plastid-containing organisms. Inactivation of GAPDH shifts the metabolic flux from glycolysis to the OPPP, producing NADPH. On the other hand, in the freshwater diatom *Asterionella* *formosa* no regulation of PRK was observed in cellular extracts [44] but GAPDH regulation appears to function much like that in *Chlamydomonas reinhardtii* via CP12 [44,45]. Additionally, putative CP12 homologs have been identified by bioinformatic means in the stramenopile *Thalassiosira pseudonana* and the haptophyte *Emiliania* *huxleyi* [18]. Crucially, the GapC1 of several marine diatoms have been shown to not be redox regulated [42] while those of several freshwater diatoms have exhibited such regulation [44,45]. Given their complex evolutionary history, acquisition of plastids by secondary endosymbiosis, and diverse metabolic lifestyles, it is not unreasonable to expect such differences in the regulation of enzymes that play such crucial roles in photosynthesis and metabolism.

In this work, we use several bioinformatic methods for predicting residues involved in functional divergence from aligned sequences in order to generate hypotheses about the molecular underpinnings of the functional shift between glycolytic GAPDH function and plastid GAPDH function for both GapC1 and GapA subgroups and to compare the two cases to identify possible convergent adaptations to the use of NADH as a cofactor versus NADPH and the specific regulatory features of plastid GAPDHs. The specific functional divergence programs we use include: 1) our recently-developed FunDi method [46] that we have shown outperforms a large number of other functional divergence predictors in simulations, 2) the program Groupsim [47] which also performed well in this study and 3) the Difference Evolutionary Trace [48–50] method. These methods are all based on widely varying methodologies. For examples, FunDi uses a phylogenetic maximum likelihood method that explicitly models functionally divergent and non-divergent sites on a particular branch of a phylogenetic tree. Difference Evolutionary Trace is also phylogeny-based, but evaluates the differences in local conservation patterns of subgroups in a phylogenetic tree. By contrast, GroupSim is not explicitly phylogenetic, but instead uses an information theoretic approach that compares within group conservation properties to between group properties while adjusting for distances between sequences in the provided alignment. Here, all three methods are applied to the comparisons between GapA sequences and cytosolic GAPDH and GapC1 and cytosolic GAPDH sequences to identify possible functionally divergent sites. Select residues predicted to be functionally divergent are analyzed in the context of known functional and regulatory data for plastid-functioning GAPDH.

In this work we also do not discriminate between sites displaying what has been called Type I and Type II functional divergence patterns [51]. Type I functionally divergent sites, also known as rate-shifting sites, are sites that are conserved in one phylogenetic sub-group but not another. In contrast Type II, or ‘conserved-but-different’, sites feature conservation (or near conservation) within both sub-groups of a phylogenetic tree but for amino acids (or groups of amino acids) with differing physico-chemical properties. While various methods exist to try and predict one versus the other type of functional divergence, all three methods used here can predict both types of sites [46]

## Materials and Methods

### Dataset Construction

All eukaryotic GAPDH sequences from the Reference Sequence (RefSeq) database of the National Center for Biotechnology Information (NCBI) annotated with the keyword glyceraldehyde-3-phosphate dehydrogenase were downloaded and clustered at the 90% identity level using UCLUST [52]. Additional non-RefSeq sequences from various microbial lineages were also assembled; including GapC1 sequences from the dataset of Takeshita and colleagues [34], cyanobacterial Gap2 sequences [9] and additional cyanobacterial GapC (cytosolic) homologs yielding a total of approximately 2000 sequences. Any 100% identical sequences were screened out, preferentially retaining any sequences currently annotated as Gap2, GapA, GapB, or GapC1 from the studies cited. This initial set of sequences was aligned with FSA [53] and trimmed using AliMask-CS (Alignment Masking with Confidence Scores), an in house alignment masking script. AliMask-CS uses the column based statistical support values output by alignment programs like FSA. A sliding window of size seven is used to calculate an average support value centred on a column. Any columns with a raw support value less than 6, an average support value less than 8, or containing more than 70% gap characters were removed from the final alignment. After these alignment and trimming steps, any sequences that were not well aligned, covered less than 75% of the trimmed alignment, or that were 100% identical to other sequences in the dataset (after trimming) were removed, leaving 490 sequences. Sequences contributing little to the overall phylogenetic/sequence diversity were then iteratively removed. To identify sequences with short terminal branch lengths, an initial phylogenetic tree was inferred using FastTree2 [54,55] with final maximum-likelihood branch length optimization using 20 gamma distributed rate categories and the JTT [56] amino acid substitution model. To avoid bias in the removal of short-branching taxa, sequences within the bottom 10th percentile of the terminal branch length distribution were identified from the non-GapC1 set and one was randomly deleted. This process was repeated until a final dataset size of 350 sequences was reached.

### Final Multiple Sequence Alignment and Phylogenetic Tree

After the reduced set of sequences was finalized, a final multiple sequence alignment was constructed using hmmalign (HMMER3 [57–59]) using an HMM profile generated from the OrthoMCL [60] seed alignment of GAPDH (OG4_10093). The alignment was then automatically trimmed using AliMask-CS as above, leaving a final alignment length of 327 amino acid positions. A final maximum-likelihood phylogenetic tree was inferred using FastTree2 selecting options to make it slightly more accurate including: slow nearest neighbour interchanges (NNIs), four rounds of sub-tree pruning and re-grafting (SPR), always optimizing all of the five relevant branch lengths during an NNI with three optimization rounds, and the slow option. Final gamma rate distributed maximum-likelihood branch length estimates were obtained as described above. The tree was displayed as arbitrarily rooted using the cyanobacterial non-Gap2, glycolytic GapC orthologs as basal taxa (all displayed trees were inferred as unrooted). In order to carry out contrast analyses of functional divergence (below) the rooted phylogenetic tree was parsed in to two alternatives, one with the GapC1 clade removed and the other with the GapA/B/2 clade removed. These contrasting trees allow for comparison of functional divergence between both sets of plastid-targeted GAPDH sequences with the glycolytic isoforms without either unduly influencing the analysis of the other.

### Testing Functional Divergence

Several different programs were used to test for sites undergoing functional divergence in plastid-targeted GAPDHs relative to cytosolic sequences. These included: FunDi [46] using QmmRAxML [61], GroupSim [47], and the Difference Evolutionary-Trace method [48–50]. Default options were used in all cases, except GroupSim where the criteria for ignoring alignment sites containing gap characters was changed in order to only ignore columns that were all gaps (none present in the tested alignments). For FunDi-based predictions, the ConsWin windowing approach of GroupSim was used as described previously [46]. To identify cases of potential convergent functional divergence between the two groups of plastid-targeted GAPDH sequences, and to avoid one group of NADPH-dependent GAPDH sequences from influencing functional divergence predictions of the other, contrast analyses were used as described above. In this technique, each group of NADPH-dependent GAPDH sequences is considered in comparison only to the cytosolic GAPDH sequences. Sites with a functional divergence score above 0.5 (FunDi, GroupSim) were considered to be functionally divergent in order to identify the maximum number of possible candidate sites for consideration. For the Difference Evolutionary-Trace method, sites are considered functionally divergent if they score within the top 20% of functionally important residues in either of the sub-groups under examination, but do not fall within the top 20% of functionally important residues when all sequences are considered in the context of the full phylogenetic tree.

### Homology Modeling

To visualize the physical context of sites predicted to be functionally divergent, a homology model was constructed using the SWISS-MODEL webserver [62–64]. Both guided and automated homology models were constructed using the *Ascophyllum* *nodosum* GapC1 sequence as input. For the guided homology models the 2PKQ (*Spinacia oleracea*) structure from the PDB was used as a template. For the fully automated mode SWISS-MODEL selected the 3E5R structure (corresponding to the cytosolic GAPDH of *Oryza sativa*) as a template. The *Ascophyllum* *nodosum* homology models based on the 2PKQ and 3E5R templates were selected for structural comparisons of NADPH/NAD+ binding.

## Results

**Figure 3:**
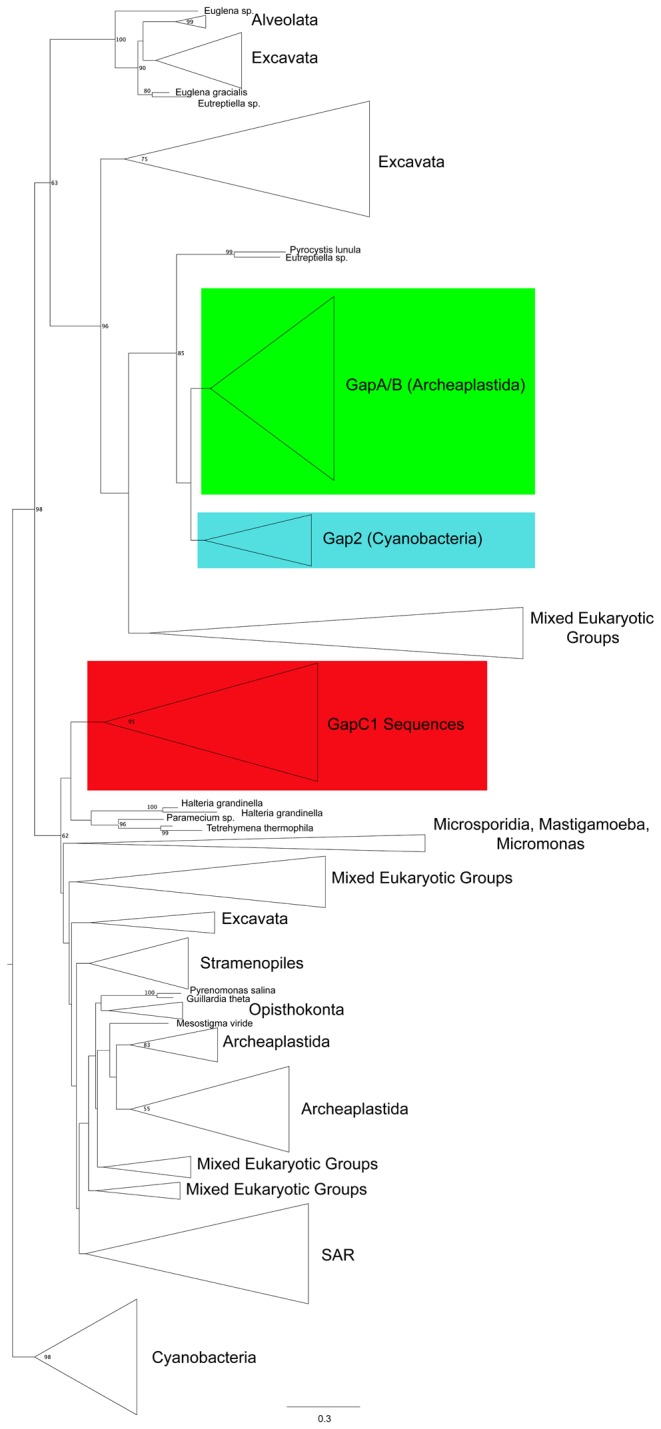
Simplified maximum-likelihood phylogenetic tree of GAPH, shown as arbitrarily rooted with cyanobacterial sequences. Cytosolic GAPDH collapsed clades are coloured in blue. Gap A/B and Gap2 are in green, with GapC1 in red. Several ciliates form the immediate outgroup to GapC1 sequences, although without bootstrap support. Only bootstrap support values above 50 are shown. Several clades, indicated as Mixed Eukaryotic Groups, belong sequences from taxa that do not form monophyletic clades. Given the many paralogs included in this analysis, and the fact that it is a single gene phylogeny, this is not unexpected. In the majority of these groups taxa are predominantly from a single supergroup with the addition of a few “rogue” taxa. The full phylogenetic tree can be found in data S1.

A dataset of 350 eukaryotic and cyanobacterial GAPDH sequences was assembled and used to infer a maximum-likelihood phylogenetic tree using FastTree2 (Figure 3). The data set was constructed to incorporate as much potential sequence diversity as possible, and as a result, the tree contains many paralogous sequences. Presumably, the various GAPDH paralogs included that are not targeted to the chloroplast bind NAD^+^ and function in glycolysis, although it is possible that some paralogs have altered functional constraints since many side-functions have been discovered for GAPDH [65–68]. In this analysis, we do not take this possibility into consideration under the assumption that many of these secondary functions will be unique to individual sequences and will not create a general functional divergence pattern over all the cytosolic homologs.

The inferred phylogenetic tree recovered GapA, GapB, and Gap2 (the cyanobacterial group of sequences most closely related to GapA/B) sequences as a monophyletic group, with Gap2 sequences branching as the sister group to GapA/B. This larger clade (Gap2, GapA, and GapB) will be referred to henceforth as the ‘green group.’ As expected the green group and GapC1 sequences fall within different locations in the overall GAPDH phylogeny. The closest branching sister taxa to the GapC1 sequences in this tree topology are several ciliates (*Tetrahymena thermophila*, *Paramecium* *tetraurelia* , and *Halteriagrandinella* ); the *Tetrahymena* and *Paramecium* sequences were those from a previous study [34] where they were used as the outgroup for determining the internal branching order within the GapC1 phylogeny. Several long-branching sequences group together basal to these sequences including microsporidians, *Bigelowiella* *natans* (Rhizaria), and the green alga *Micromonas* *pusilla* . Based on the long branches and lack of robust support in the phylogeny, it is likely that many of these sequences represent highly divergent paralogs and their position and grouping together may be the result of long-branch attraction, although this possibility was not further investigated in this study.

### Sites Predicted to be Functionally Divergent

In order to evaluate the predictions made by FunDi, and to provide support for cases of possible convergent evolution, we discuss a selection of sites predicted to be functionally divergent within their structural and functional context. A wealth of functional and regulatory data has been accumulated, at the residue and domain level, for both cytosolic and plastid-functioning GAPDH enzymes, particularly sites involved in differentiating NADH and NADPH, as well as the CP12 mediated regulation of plastid-targeted GAPDH in the Archaeplastida. Predictions made by FunDi constitute hypotheses about sites that may play a role in functional divergence, and for sites divergent in both groups, possible cases of convergent evolution. While some of these predictions (discussed below) have experimental evidence based on previous work, others may be useful for further experimental validation and follow-up.

All three predictors of functional divergence identified a number of alignment positions with significant functional divergence scores for both the green and GapC1 groups versus cytosolic GAPDH. Table 1 lists the number of sites predicted to be functionally divergent in the green group only, GapC1 only, and those predicted as functionally divergent in both groups for each of the prediction methods used. FunDi using QmmRAxML for site-likelihood calculation predicts the largest number of sites while the Difference Evolutionary Trace Method predicts the least. To place the predictions of functional divergence into context, and to describe the differences in performance between FunDi, GroupSim, and the Difference Evolutionary-Trace Method, we gathered data from many functional and structural studies of GAPDH, paying particular attention to NADPH/NAD+ binding and the regulation of function by CP12. All position numbering is according to the structure of *Bacillus stearothermophilus* [69] and the other structures from PDB used here. All sites discussed in their structural/functional context below are summarized in Table 2. Unless otherwise noted all positions were predicted to be functionally divergent by FunDi.

**Table 1:** Number of sites predicted to be functionally divergent in each of the groups in question, or in both groups, for each of the classifiers used.

	Green Group Only	GapC1 Only	Shared
FunDi	69	26	20
GroupSim	37	4	2
Difference Evolutionary Trace	17	15	7

**Table 2:** Sites predicted to be functionally divergent by at least one of the three programs (FunDi, Difference Evolutionary Trace, GroupSim).

Site	FunDi	Difference Evolutionary Trace	GroupSim	Evidence
Val28	Both			Proximity
Val29	GapA			Proximity
Asp32	C1			Mutagenesis/Crystallography
Thr33	GapA			Mutagenesis/Crystallography
Gly34	C1			Mutagenesis/Crystallography
Gly35	GapA			Mutagenesis/Crystallography
Val74	Both			Proximity
Ser75	Both			Proximity
Asp76	Both	GapA	GapA	Proximity
Arg77	Both	Both		Mutagenesis/Crystallography
Asn79		Both		Proximity
Asn146	C1	C1	C1	Proximity
Cys153	C1		C1	Proximity
Arg183	GapA	GapA	GapA	Crystallography
Ser188	Both		Both	Mutagenesis/Crystallography
Arg191			GapA	Crystallography
Arg195		GapA	GapA	Crystallography

Indicated is whether site was predicted to be divergent in the GapA Group, GapC1, or both. In the evidence column the type of evidence supporting the hypothesis of functional divergence is indicated. Most residues with experimental evidence are combined mutagenesis experiments, often with crystallographic support as well. Other sites are merely in close proximity to studies residues and are marked accordingly.

### Loop positions 32-35

Aspartate 32 (Figure S1) is one of the residues implicated in the binding of both NAD^+^ and NADPH in the photosynthetic form of GAPDH [70]. When NAD^+^ is bound, Asp32 forms a hydrogen bond with the 2’-hydroxyl group of the adenosine in NAD^+^ [71], presumably in the same manner as in cytosolic GAPDH. Alternatively, when NADPH is bound, it rotates away from the cofactor thereby preventing steric clashes [16,17]. This position is conserved in chloroplast GAPDH sequences as well as Gap2 sequences and is highly conserved as an aspartate residue in cytosolic GAPDH sequences. In the GapC1 plastid-targeted sequences this site is not as well conserved with amino acids such as glutamate, serine, threonine, and alanine all observed, and predicted to be functionally divergent, but only in that clade. In the sequences where aspartate has been substituted with glutamate, it is likely that glutamate is performing the same function. When non-acidic residues at position 32 are observed, it is likely that other residues on the flexible loop where this site is located are involved in NAD^+^ binding in those sequences.

There are several sites that lie in close proximity to the conserved aspartate 32 residue, such as valine 28 and 29, threonine 33, and glycine 34 and 35. Valine 28 was predicted to be functionally divergent in both groups while valine 29 was predicted only among the green group. Thr33 and Gly35 were only predicted as functionally divergent in the green group and Gly34 only in the GapC1 clade. Previous experimental work in the NAD^+^-binding GAPDH of the thermophilic *Eubacterium* *Bacillus stearothermophilus* [16,70,72] involved mutating several of these residues (33, 34, and 35), which had previously been described as a NAD^+^-binding ‘fingerprint’ [73,74], to their counterparts in NADPH-dependent GAPDH to investigate their role in discrimination between the two co-enzymes. While mutating these three residues, even in combination with other important S-loop residues described below, to their plastid-GAPDH specific residues did not recapitulate plastid-GAPDH NADPH-binding affinity, there was some effect [72]. In this mutant dual-coenzyme specificity was observed, but with a marked shift in preference for NAD^+^ binding. In this case the effect of other residues not mutated to their chloroplastic counterparts interfered with NADP-binding [72]. It is important to consider that those mutagenesis experiments were conducted on the cytosolic GAPDH of a thermophilic bacteria, expressed in *E. coli*. Other amino acids are likely to affect the overall structure of this protein, making it a poor model. In other studies mutating threonine 33 alone to alanine had a marked impact on NADPH-binding affinity [16]. While mutating these residues alone is not sufficient to convert a NADH-binding preference to NADPH-binding, at least some of these residues clearly have an impact on NADPH-binding affinity.

### Arginine 77

Arginine 77 (Figures 3 and 4) is located in the “cleft” between GAPDH monomers in the tetrameric complex along one of several flexible loop regions, physically near to threonine 33 and aspartate 32 and is predicted to be functionally divergent by the FunDi method in both the green and GapC1 groups. In one wild-type crystal structure [17] at 3.0 Å resolution this residue was highly disordered; however, in a second, more recent, 2.0 Å resolution crystal structure, Arg77 had a high degree of order and was positioned to form a salt-bridge with the 2’-phosphate of NADPH [16]. The increased resolution of the more recent structure, combined with the fact that a conserved arginine generally is involved in binding the 2’-phosphate of NADPH in other NADPH-binding enzymes with Rossman folds [75] provided increased evidence that Arg77 is involved in NADPH binding.

As well as stabilizing the 2’-phosphate of NADPH via a salt bond, Arg77 also plays an important role in the CP12-mediated regulation of NADPH-dependent activity in GAPDH (Figure S2). When CP12 enters in to the “cleft” region between R-axis related monomers, Arg77 moves away from potential interactions with the 2’-phosphate of NADPH and swings towards CP12 where it is thought to interact with the negatively charged residues of the regulatory peptide [14,16,19,76,77]. In the multiple sequence alignment, arginine is strictly conserved in both the GapA and GapC1 clades, but is not conserved among cytosolic GAPDHs, although arginine and lysine are the two most commonly occurring amino acids at that position in the latter sequences. This is likely a case of convergent Type I (rate shifting) functional divergence where the evolutionary rate has shifted between groups through fixation of an arginine and subsequent purifying selection to maintain this residue due to its important role in both regulation and binding of NADPH.

### Serine 188

Serine 188 (Figures 3 and 4) is located on the S-Loop of GAPDH where it hydrogen bonds with the 2’-phosphate group of NADPH bound to the monomer located across the R-axis of symmetry [16,17,70,72]. When NAD^+^ is bound, as in the down-regulated form of plastid-targeted GAPDH, Ser188 instead forms hydrogen bonds with available water molecules and potentially with Asn39 of the opposite, R-related subgroup [16]. This position is almost universally conserved as a serine within the green group and the GapC1 sequences, with a handful of substitutions to alanine, and some to threonine in the latter clade, while prolines predominate at this position in cytosolic GAPDH. This position was predicted to be functionally divergent in both the green group and GapC1.

In mutants with Ser188 substituted by an alanine [16] there is a reduced preference for NADPH over NAD^+^ and a phenotypic difference characterized by a “loosened” and enlarged conformation due to the loss of the Ser188 interaction with bound NADPH. This is probably not the case for Ser188Ala substitutions observed in some GapC1 sequences due to the presence of additional serine or threonine residues at positions at neighbouring sites on the S-loop such as 187 or the insertion between 188 and 189 in GapC1 relative to the GapA sequences. Position 187 is often a serine or threonine in the GapC1, compared to a conserved alanine in the green group, and there is no strict conservation among the other sequences in the alignment. It has been hypothesized previously that the alanine found at position 187 in GapA-type plastid-targeted GAPDHs reduces steric clash with the 2’phosophate of NADPH and allows serine 188 to form the necessary hydrogen bond [17,70,72,78,79]. This position was also predicted to be functionally divergent in both the GapC1 and the GapA group.

### Other important S-loop Residues Near Serine 188

Among the canonical NADPH-dependent GAPDH sequences of plants, the S-loop contains several conserved arginine residues (183, 191, and 195) that, together with Ser188, are thought to be important residues involved in interactions with CP12 [76] (Figure S2). Due to this excess of positively-charged residues, and because the regulatory region of CP12 and the C-terminal extension of GapB contain an excess of negatively-charged residues, it has been hypothesized that there is an important interaction between the two, especially given the “enlarged” phenotype seen in Ser188Ala mutants. Several of these residues on the S-loop were predicted to be functionally divergent. Only arginine 183 was predicted to be functionally divergent in the GapA group, but not in the GapC1 sequences. They are all strictly conserved arginine residues in the GapA GAPDH sequences and not strictly conserved in cytosolic GAPDH’s, although lysine and arginine are frequently observed and there are conserved arginine residues very near (often at neighbouring positions) on the S-loop.

### Other Sites in the Coenyzme Binding Domain

There are other sites within the coenzyme-binding domain predicted to be functionally divergent, including sites predicted in both the GapC1 sequences and the GapA group. While we cannot conclusively assign functional roles to all of these residues based on experimental studies, some are located in close proximity to the residues described above, including four residues all located on the same loop as arginine 77 (Val74, Ser75, and Asp76). Because Arg77 plays an important role in both coenzyme binding and the thioredoxin-mediated regulation of GAPDH during light/dark cycles, residues nearby may also play direct or indirect roles in regulation, or are coevolving. Residues 74, 75, and 76 are almost universally conserved within the GapA group with a V–S-[NDT] motif, while these three positions are not conserved among cytosolic GAPDH sequences. The chromalveolate GapC1 sequences are less strongly conserved, with a tendency for a [ST]-[HA]-T motif, although there are some exceptions. It is possible that these positions may influence GapC1 regulation. The motif for *Odontella* for instance, which lacks CP12 regulation [42], is a very divergent S-R-C while for *Ascophyllum*, which does appear to have CP12 regulation, it is the more canonical GapC1 motif of S-A-T. Based on the sequence conservation patterns in these residues it does not appear as if there is a strong argument for convergence between the GapA group and GapC1 sequences. Nevertheless this region may still be important for CP12-mediated regulation, and the differences observed within the GapC1 sequences may partially explain some of the observed regulatory differences. The accumulation of more regulatory and sequence data will be needed to test these speculations. All three positions were predicted to be functionally divergent in both groups by FunDi.

### Comparison with Evolutionary-Trace and GroupSim

We compared the functional divergence predictions made by FunDi for residues previously described to play important functional roles with those of both the Difference Evolutionary-Trace Method and Group, Sim. Neither aspartate 32, nor any of the nearby positions described above, were predicted to be functionally divergent by either of the latter two methods. The Difference Evolutionary-Trace method also predicted arginine 77 to be functionally divergent in both groups of plastid-targeted GAPDH sequences. Aspartate 76 was predicted to be functionally divergent by both the Evolutionary Trace and Group, Sim methods, as it was by FunDi, but only within the GapA group. Arginine 195 was predicted by both the Difference Evolutionary-Trace method and GroupSim in the GapA group, but not FunDi. Asparagine 79, also within a region thought to be important in both function and regulation between the two groups of plastid-targeted sequences, was only predicted to be functionally divergent by Difference Evolutionary-Trace. Serine 188 was predicted to be functionally divergent by GroupSim in both groups, as it was by FunDi, while arginine 191 was predicted only by GroupSim (GapA only) and not any other method.

## Discussion

While there is substantial overlap in the sites predicted to be functionally divergent in NADPH-dependent GAPDH sequences by all of the predictors used in this work, especially among sites that have been previously linked with functional differences, there are also clear discrepancies between them. FunDi predicted the most sites and also consistently predicted more known functional sites as being functionally divergent consistent with its better performance assessed by simulation in our previous work [46]. Serine 188 and arginine 77 were both predicted by FunDi while only GroupSim predicted the former and the Difference Evolutionary Trace method the latter. The only known functionally divergent residues missed by FunDi, with either likelihood calculation method, were two of the potentially regulatory arginines (191 and 195) on the S-loop.

There also appears to be evidence for convergent evolution between cyanobacterial derived GapA sequences and the GapC1 clade. Not only were 28 residues predicted to be functionally divergent in both groups between all of the prediction methods tested here, but also many of the key positions converged on the same or similar amino acid residues. Arginine 77 and serine 188 for instance, previously identified in crystallographic and mutagenesis studies as being key residues for the discrimination between NADPH and NAD^+^, converged on the same residue in both GapA and GapC1. Serine 188 in particular is striking, as the homologous position in cytosolic GAPDHs is generally a conserved proline.

Residues of interest are also located near to arginine 77 (positions 74, 75, and 76) predicted by FunDi with QmmRAxML as being functionally divergent in both groups of NADPH-dependent GAPDH sequences, along with position 79, which was predicted to be divergent in both groups only by GroupSim. To our knowledge, no experimental work has suggested a functional role related to the differences between NADH- and NADPH-dependent GAPDH sequences for these sites. However, their level of conservation in these groups suggests that, at the very least, they contribute to the physical and chemical environment of arginine 77 and we predict that they may be involved in interactions with CP12. These residues, while functionally divergent in both plastid-derived GAPDH clades, are not an example of convergent evolution as they feature very different sequence motifs in the two groups. For GapC1, fingerprint regions such as this where some variability is observed among lineages may help explain the apparent differences observed in GapC1 regulation between GapC1-containing taxa (see the Introduction) [41], although more functional and sequence data is required to test this hypothesis.

The foregoing analyses showcases the utility of FunDi for the identification of key residues involved in functional divergence, especially when analyzing large and phylogenetically complex datasets. While no single classifier identified all of the important specificity-determining sites with experimental validations, FunDi predicted the most and was markedly better at identifying cases of convergent evolution. Based on previous simulation results [46], we can be confident that this improved true positive prediction rate is not generally achieved at the expense of a drastic increase in false positives. It is also probable that many of the sites predicted to be functionally divergent without experimental validation, are in fact functionally divergent and/or are co-evolving with functionally divergent residues or are responsible for maintaining appropriate protein folding dynamics, stability, or function. This example serves to illustrate how FunDi can be used not only to make inferences about functional changes in protein families, but it can also be used to guide follow-up experimental investigations (e.g. mutagenesis experiments) of such sites to enhance understanding of the precise nature of the functional changes.

## Supporting Information

Figure S1 **Key residues for co-enzyme discrimination identified by previous experimental work.**
Cartoon representation of O and R subunits from PDB 2PKQ are shown as in Figure 1. Asp32 and Thr33 located behind bound NADP molecule, with Asp32 rotated away to reduce steric clash with 2’-phosphate of NADP. Ser188 and Arg77 are shown in positions to interact with 2’-phosphate of bound NADP.
(TIF)

Figure S2 **The “Cleft” and CP12 binding region between monomers in the A2B2 tetramer of GAPDH from spinach (2PKQ)**.
The C-terminal extension of GapB, homologous to C-terminal regulatory region of CP12 and important conserved arginine residues are indicated. Bound NADP^+^ is coloured by atom type but is shown as transparent as are the P and R monomers. Conserved arginines, important for CP12 interaction are shown from both monomers.
(TIF)

Data S1 (ZIP)
