# Supplementary figures and images for: Functional Divergence and Convergent Evolution in the Plastid-Targeted Glyceraldehyde-3-Phosphate Dehydrogenases of Diverse Eukaryotic Algae
Source: PLoS One. 2013 Jul 30;8(7):e70396. doi: 10.1371/journal.pone.0070396 (PMC3728087; doi:10.1371/journal.pone.0070396)

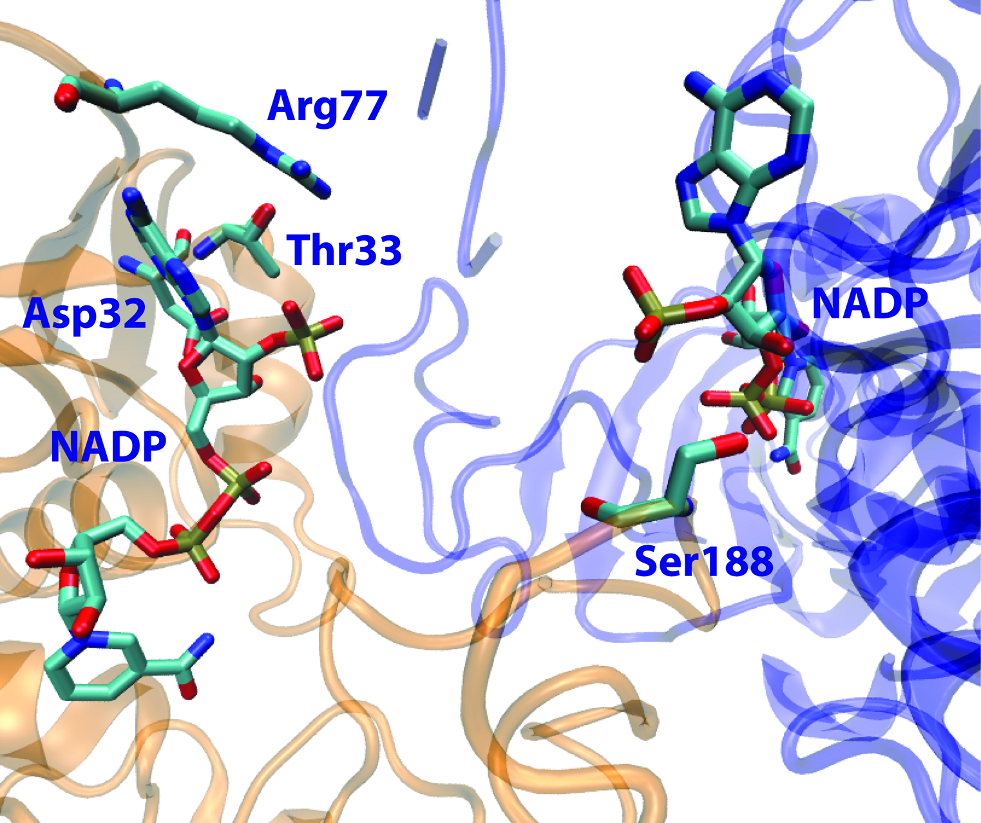

Supplement: Figure S1 — Key residues for co-enzyme discrimination identified by previous experimental work. Cartoon representation of O and R subunits from PDB 2PKQ are shown as in Figure 1. Asp32 and Thr33 located behind bound NADP molecule, with Asp32 rotated away to reduce steric clash with 2’-phosphate of NADP. Ser188 and Arg77 are shown in positions to interact with 2’-phosphate of bound NADP. (TIF) [file pone.0070396.s001.tif]

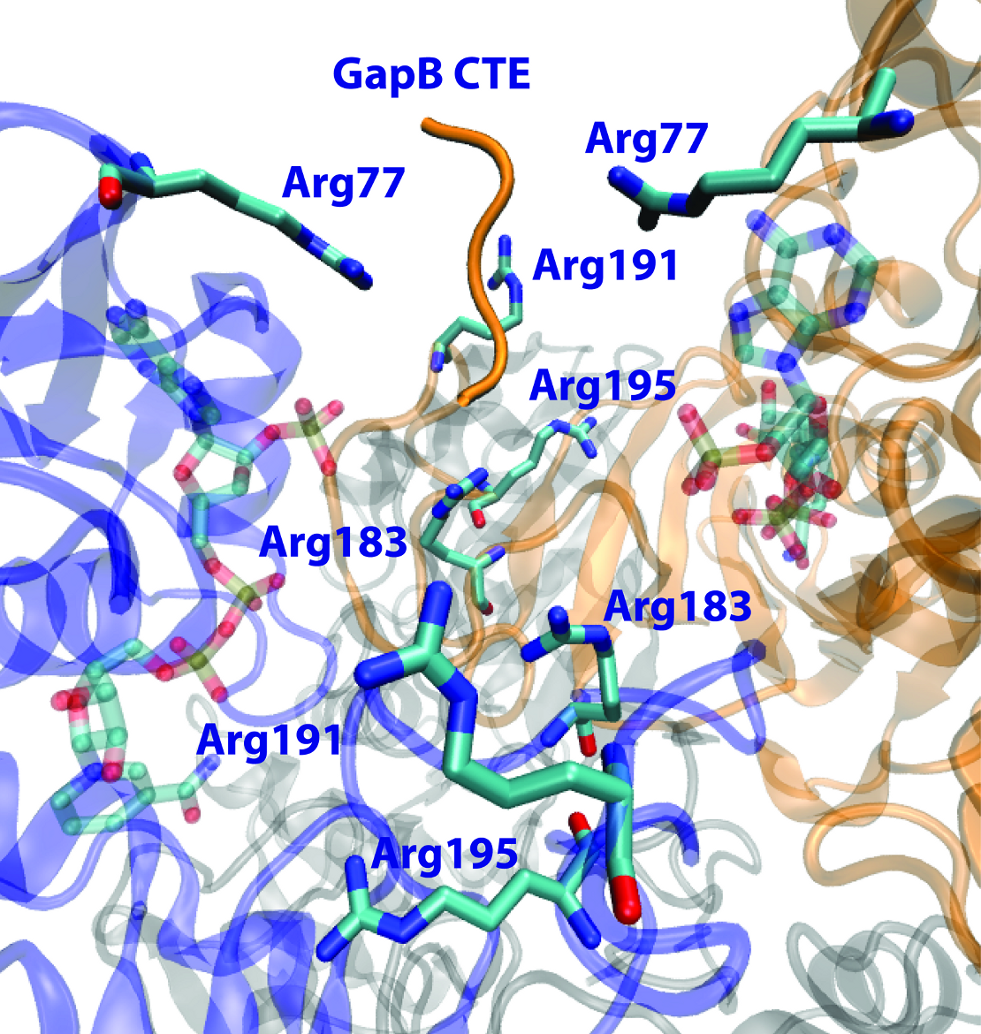

Supplement: Figure S2 — The “Cleft” and CP12 binding region between monomers in the A2B2 tetramer of GAPDH from spinach (2PKQ). The C-terminal extension of GapB, homologous to C-terminal regulatory region of CP12 and important conserved arginine residues are indicated. Bound NADP+ is coloured by atom type but is shown as transparent as are the P and R monomers. Conserved arginines, important for CP12 interaction are shown from both monomers. (TIF) [file pone.0070396.s002.tif]
